# Supplementary material for: Third-Generation Antipsychotics and Lurasidone in the Treatment of Substance-Induced Psychoses: A Narrative Review
Source: Healthcare (Basel). 2024 Jan 29;12(3):339. doi: 10.3390/healthcare12030339 (PMC10855531; doi:10.3390/healthcare12030339)
Supplement: Supplementary file 1 [file healthcare-12-00339-s001.zip › Table S1.pdf]

Table S1. MAIN FINDINGS OF RETRIEVED STUDIES IN ARIPIPRAZOLE.

REFS	PSICHIATRIC DIAGNOSIS	KEY CONCEPT	POPULATIO N	SUBSTANCE-RELATED DIAGNOSIS	SAMPLE	RESULTS
**Leite et al., 2008**	nd	Aripiprazole's efficacy in treating motor hyperactivity from dopaminergic and glutamatergic drugs, aimed at schizophrenia and psychostimulant abuse management.	nd	Psychostimulants	Animal	Aripiprazole (0.1-1 mg/kg) attenuated reinforcing properties induced by psychotomimetic and psychostimulants
**Sørensen et al., 2008**	nd	Aripiprazole's impact on cocaine's D2 receptor effects and self-administration in mice	nd	Cocaine	Animal	Aripiprazole (0.4 mg/kg) blocks acute self-administration of cocaine and is not self-administered in mice.
**Rolland et al., 2013**	Cannbis induced psychosis	Aripiprazole mitigates Cannabis-Induced Psychosis in high-risk patients without decreasing cannabis use, indicating harm-reduction potential.	1	Cannabis	Human	Positive outcomes from the administration of 10 mg/die of aripiprazole, even though there was no observed decrease in the frequency of smoking
**Desseilles et al., 2008**	Schizophrenia in cannabis abusers	Aripiprazole aids in ceasing cannabis use and improving symptoms in a schizophrenic patient, indicating potential for dual diagnosis treatment	1	Cannabis	Human	Aripiprazole 10 mg/die in reducing cannabis intake in a schizophrenic
**Feltenstein et al., 2007**	nd	Aripiprazole reduces cocaine-seeking in rats without affecting other behaviors, showing promise for cocaine dependency relapse prevention	nd	Cocaine	Animal	Aripiprazole (0.25-1 mg/kg) blocks reinstatement of cocaine seeking in an animal model of relapse

**Thurstone et al., 2023**	Psychosis and co-occuring cannabis use disorders	Aripiprazole faster than risperidone in treating psychosis and cannabis disorder in hospitalized adolescents	110	Cannabis	Human	Adolescents hospitalized for co-occurring psychosis and cannabis use disorder showed rapid reduction in acute psychotic symptoms for aripiprazole (15 mg/die)compared with risperidone
**Futamura et al., 2010**	nd	Aripiprazole mitigates methamphetamine effects in mice, indicating potential for treating stimulant psychosis and schizophrenia relapse.	nd	Methamphetamine	Animal	Treatment with aripiprazole (0.1-1 mg/kg) from days 10 to 14 during withdrawal from MAP administration attenuated the effect of MAP pretreatment, enhancing the motor response to a challenge dose of stimulant 3 days after the aripiprazole preparation
**Farnia et al., 2014**	Amphetamine induced psychosis	Risperidone and aripiprazole are both effective in treating amphetamine-induced psychosis: risperidone is better for positive symptoms and aripiprazole for negative symptoms	45	Amphetamine	Human	Risperidone appeared more promising than aripiprazole (15 mg/die) in positive symptoms aripiprazole exhibited greater potential in reducing negative symptoms.
**Wang et al., 2016**	Methamphetamine induced psychosis	Both aripiprazole and risperidone effectively treat methamphetamine-associated psychosis,.	42	Methamphetamine	Human	Both aripiprazole (5-15 mg/die) and risperidone may be effective for the treatment of patients with METH-associated psychosis. Aripiprazole efficacy, may be comparable to risperidone but risperidone shows better patient retention and less adverse effects compared to aripiprazole.
**Skryabin et al., 2021**	Schizophreniaand substance use disorders (polysubstance users)	Aripiprazole alleviates psychotic symptoms in polysubstance abusers and reduce cocaine dependence	90	Polysubstances	Human	Aripiprazole (up 20 mg/die) and quetiapine exhibit comparable efficacy in alleviating psychotic symptoms., aripiprazole demonstrated superior effectiveness in mitigating cocaine dependence

**Sulaiman et al., 2013**	Schizophrenia and co-occurrence Methamohetamine dependence	Aripiprazole aids in treatment adherence and lessens psychosis in methamphetamine dependency, safe but not enhancing abstinence	37	Methamphetamine	Human	Aripiprazole (5-10 mg/ide) was no more effective than placebo in maintaining abstinence from methamphetamine use. However, it facilitated treatment retention and reduced the severity of psychotic symptoms. Aripiprazole was found to be generally safe and well tolerated
**Beresford et al., 2005**	Schizophrenia with comorbid cocaine dependence	Aripiprazole potentially reduces cocaine and alcohol cravings in schizophrenia with cocaine dependence, suggesting further research need	10	Cocaine	Human	Aripiprazole effects 15 mg/die in lowering both desire for and the use of cocaine in comorbid schizophrenic subjects
