# Supplementary material for: Third-Generation Antipsychotics and Lurasidone in the Treatment of Substance-Induced Psychoses: A Narrative Review
Source: Healthcare (Basel). 2024 Jan 29;12(3):339. doi: 10.3390/healthcare12030339 (PMC10855531; doi:10.3390/healthcare12030339)
Supplement: Supplementary file 1 [file healthcare-12-00339-s001.zip › Table S2.pdf]

Table S2. MAIN FINDINGS OF RETRIEVED STUDIES IN CARIPRAZINE.

REFS	PSICHIATRIC DIAGNOSIS	KEY CONCEPT	POPULATION	SUBSTANCE-RELATED DIAGNOSIS	SAMPLE	RESULTS
**Roman et al., 2013**	nd	Cariprazine, aripiprazole, and bifeprunox show potential in reducing cocaine reward and preventing relapse in cocaine addiction	nd	Psychostimulants	Animal	Cariprazine (0.03–1 mg/kg) demonstrates anti-abuse potential in rats
**Jordan et al., 2020**	nd	Positive role of cariprazine in cocaine reward in rodens	nd	Cocaine	Animal	Cariprazine (1-5mg·kg^{-1}) attenuates cocaine reward and relapse in rodents.
**Rodriguez Cruz et al., 2021**	Schizophrenia in polysubstance abuser	Switching from Haloperidol to Cariprazine significantly improved symptoms and reduced substance abuse in a schizophrenia patient with a history of drug abuse.	1	Polysubstances	Human	Cariprazine (4.5 mg-die) ameliorates positive, negative, and cognitive symptoms in a schizophrenic patient with an extensive history of substance abuse
**Ricci et al., 2022**	Methamphetamine induced psychosis	Cariprazine effectively reduced psychotic and addictive symptoms in a methamphetamine-induced psychosis case, indicating its potential therapeutic value	1	Methamphetamine	Human	Potential effect of cariprazine (4.5 mg/die)on a drug naïve patient with persistent psychotic symptoms induced by methamphetamine for the first time.
**Truong et al., 2022**	Methamphetamine induced psychosis	Cariprazine effectively reduced cravings and use in methamphetamine use disorder, indicating its potential as a novel treatment.	2	Methamphetamine	Human	Cariprazine (1.5mg/die-3 mg /die) showed global functional improvement and reduction in methamphetamine cravings
**Montes et al., 2021**	Psychosis in methamphetamine, mephedrone and cocaine abuse	Cariprazine is efficacy in polysubstance-induced psychosis	1	Cocaine, Mephedrone, Methamphetamine	Human	Cariprazine (3 mg/die)effectively alleviated positive symptoms in a 31-year-old male who was HIV positive and had a history of abusing methamphetamine, mephedrone, cocaine, and alcoho
**Gentile et al., 2022**	Cannabis induced psychosis	Cariprazine shows positive effects in treating co-morbid psychosis and cannabis Intake	1	Cannabis	Human	Administration of cariprazine (3 mg/die) led to an improvement in both negative and positive symptoms and resulted in a reduction in cannabinoid intake.
