# Supplementary material for: Third-Generation Antipsychotics and Lurasidone in the Treatment of Substance-Induced Psychoses: A Narrative Review
Source: Healthcare (Basel). 2024 Jan 29;12(3):339. doi: 10.3390/healthcare12030339 (PMC10855531; doi:10.3390/healthcare12030339)
Supplement: Supplementary file 1 [file healthcare-12-00339-s001.zip › Table S3.pdf]

Table S3. MAIN FINDINGS OF RETRIEVED STUDIES IN BREXPIPRAZOLE.

REFS	PSICHIATRIC DIAGNOSIS	KEY CONCEPT	POPULATIO N	SUBSTANCE RELATED DIAGNOSIS	SAMPLE	RESULTS
**Nickols et al., 2023**	nd	Brexpiprazole as a Dopamine Partial Agonist in Opioid Dependence Treatment	nd	Opioids	Animal	Brexpiprazole (0.5 mg/kg) is modulator of dopamine-dependent behaviors during opioid use and withdrawal
**Kung et al., 2022**	Psychosis during cannabis withdrawal	Efficacy of brexpiprazole in cannabis withdrawal-induced psychosis	1	Cannabis	Human	Brexpiprazole is effective in treating psychosis during cannabis withdrawal in a 25-year-old individual at a daily dosage of 3 mg
