# Supplementary material for: Third-Generation Antipsychotics and Lurasidone in the Treatment of Substance-Induced Psychoses: A Narrative Review
Source: Healthcare (Basel). 2024 Jan 29;12(3):339. doi: 10.3390/healthcare12030339 (PMC10855531; doi:10.3390/healthcare12030339)
Supplement: Supplementary file 1 [file healthcare-12-00339-s001.zip › Table S4.pdf]

Table S4. MAIN FINDINGS OF RETRIEVED STUDIES IN LURASIDONE.

REFS	PSICHIATRIC DIAGNOSIS	KEY CONCEPT	POPULATION	SUBSTANCE RELATED DIAGNOSIS	SAMPLE	RESULTS
**Ricci et al., 2022**	Cannabis induced psychosis	Efficacy of lurasidone in cannabis induced psychosis	4	Cannabis	Human	Lurasidone showed improvements in both positive and negative symptomatology in four patients. The dosage of lurasidone ranged from 74 mg to 111 mg
**Mole et al., 2022**	Cannabis, LSD Induced psychosis	Efficacy of lurasidone in a polyabuser patient with psychotic symptoms	1	Cannabis<br>LSD<br>Alcohol	Human	Beneficial use of lurasidone (40 mg/die) in a 14-year-old with LSD, cannabis induced psychosis
