# Supplementary material for: Third-Generation Antipsychotics and Lurasidone in the Treatment of Substance-Induced Psychoses: A Narrative Review
Source: Healthcare (Basel). 2024 Jan 29;12(3):339. doi: 10.3390/healthcare12030339 (PMC10855531; doi:10.3390/healthcare12030339)
Supplement: Supplementary file 1 [file healthcare-12-00339-s001.zip › Table S5.pdf]

Table S5. Characteristics of antipsychotic drugs resulted to be used in Substance Induced Psychosis.

ANTIPSYCHOTIC DRUG	MECHANISM OF ACTION	THERAPEUTIC REGIMEN	CURRENT EVIDENCE	SIDE EFFECTS
**Aripiprazole**	Partial agonism at dopamine D2 and serotonin 5-HT1A receptors; Antagonism at serotonin 5-HT2A receptors; Blockade of serotonin type 5-HT2C and 7 receptors	Orally: 15-30 mg/day; available as a depot formulation (400 mg fl/28 days)	Significant improvement in positive and negative symptoms, reduction in substance use and craving (cocaine and cannabis)	Better safety profile than other AP in terms of extrapyramidal side effects, sedation, and metabolic changes. Akathisia and agitation are reported (Wang et al., 2016)
**Cariprazine**	Partial agonist at the dopamine (DA) D2 and D3 receptors and serotonin 5-HT1A receptors, and as an antagonist at the 5-HT2B receptors.	Orally: 1.5-3 mg/day;	Improvement in substance induced psychosis (psychostimulant, cocaine and methamphetamine)	Good safety profile. No reported side effects. Only insomnia treated with benzodiazepines (Ricci et al.,2022)
**Brexpiprazole**	Partial agonism of 5-HT_{1A} and D_2 receptors and antagonism of 5-HT_{2A}. Brexpiprazole binds with high affinity to numerous monoaminergic receptors encompassing D2, D3, 5-HT1A, 5-HT2A, 5-HT2B, and 5-HT7	Orally: 2-4 mg/day;	Improvement cannabis withdrawal psychosis. Modulator of dopamine-dependent behaviors during opioid use in rats	Good safety profile. No reported side effects.
**Lurasidone**	Full antagonist at dopamine D2 and serotonin 5-HT2A and 5-HT7 receptors	Orally: 40-160 mg/day;	Improvement in cannabis induced psychotic symptoms and LSD	Good safety profile. Only sedation reported (Ricci et al., 2022)
