# Supplementary material for: Third-Generation Antipsychotics and Lurasidone in the Treatment of Substance-Induced Psychoses: A Narrative Review
Source: Healthcare (Basel). 2024 Jan 29;12(3):339. doi: 10.3390/healthcare12030339 (PMC10855531; doi:10.3390/healthcare12030339)
Supplement: Supplementary file 1 [file healthcare-12-00339-s001.zip › Table S6.pdf]

TABLE S6. Receptor-binding profile. Antagonism and inverse agonism are indicated by neutral color whereas partial agonism by yellow. The number of crosses is correlated to binding affinity. 100 < Ki < 1000: + weak association. 10< Ki < 100: ++ moderate association. 1< Ki < 10: +++ strong association. 1 > Ki: ++++ very strong association.

	D_1	D_2	D_3	D_4	H_1	H_2	H_3	5-HT_{1A}	5-HT_{1B}	5-HT_{2A}	$5HT_{2B}$	5-HT_{2C}	5-HT_6	5-HT_7	M_1	M_3	α_1	α_{2a}	α_{2b}	α_{2c}
**ARIPIPRAZOLE**		+++	+++	+	++			+++	+	++	++++	++	+	++			++	++	++	++
**LURASIDONE**	+	+++						+++		+++		+		++++			++	++		+++
**BREXPIPRAZOLE**	+	++++	+++	+++	++			++++	++	++++	+++	+	++	+++			+++	++	++	++++
**CARIPRAZINE**		++++	++++		++			+++		++	++++	+		+			+			
